# Remodeling of Actin Cytoskeleton in Mouse Periosteal Cells under Mechanical Loading Induces Periosteal Cell Proliferation during Bone Formation

**DOI:** 10.1371/journal.pone.0024847

**Published:** 2011-09-14

**Authors:** Daisuke Sakai, Isao Kii, Kazuki Nakagawa, Hiroko N. Matsumoto, Masateru Takahashi, Suguru Yoshida, Takamitsu Hosoya, Kazuo Takakuda, Akira Kudo

**Affiliations:** 1 Department of Biological Information, Tokyo Institute of Technology, Yokohama, Japan; 2 Division of Biosystems, Department of Biodesign, Institute of Biomaterials and Bioengineering, Tokyo Medical and Dental University, Tokyo, Japan; 3 Laboratory of Chemical Biology, Graduate School of Biomedical Science, Institute of Biomaterials and Bioengineering, Tokyo Medical and Dental University, Tokyo, Japan; Ohio State University, United States of America

## Abstract

**Background:**

The adaptive nature of bone formation under mechanical loading is well known; however, the molecular and cellular mechanisms *in vivo* of mechanical loading in bone formation are not fully understood. To investigate both mechanisms at the early response against mechanotransduction *in vivo*, we employed a noninvasive 3-point bone bending method for mouse tibiae. It is important to investigate periosteal woven bone formation to elucidate the adaptive nature against mechanical stress. We hypothesize that cell morphological alteration at the early stage of mechanical loading is essential for bone formation *in vivo*.

**Principal Findings:**

We found the significant bone formation on the bone surface subjected to change of the stress toward compression by this method. The histological analysis revealed the proliferation of periosteal cells, and we successively observed the appearance of ALP-positive osteoblasts and increase of mature BMP-2, resulting in woven bone formation in the hypertrophic area. To investigate the mechanism underlying the response to mechanical loading at the molecular level, we established an *in-situ* immunofluorescence imaging method to visualize molecules in these periosteal cells, and with it examined their cytoskeletal actin and nuclei and the extracellular matrix proteins produced by them. The results demonstrated that the actin cytoskeleton of the periosteal cells was disorganized, and the shapes of their nuclei were drastically changed, under the mechanical loading. Moreover, the disorganized actin cytoskeleton was reorganized after release from the load. Further, inhibition of onset of the actin remodeling blocked the proliferation of the periosteal cells.

**Conclusions:**

These results suggest that the structural change in cell shape via disorganization and remodeling of the actin cytoskeleton played an important role in the mechanical loading-dependent proliferation of cells in the periosteum during bone formation.

## Introduction

The material composition and structure of bone determine the strength and elasticity of bone to tolerate a load, and the modulate loads on bone strengthen its structure. This adaptation is carried out by the cellular machinery of bone remodeling. Bone remodeling functions in damage repair; and therefore, the adaptation of structure to loading is anticipatory damage prevention [1].

For investigation of the function of this adaptive nature, various apparatuses of mechanical loading have been developed. For example, Turner *et al.* developed a rat tibia model under 4-point bending [2]; and Torrrance *et al.*, a rat ulna model under axial compression [3]. These models were employed to study loading-related bone formation on intact bones without surgical intervention. In further development of model systems concerning the timeline factors, which are important for elucidating the nature of the mechanotransduction system, noteworthy investigations have been conducted by Robling *et al.* [4], [5] and Srinivasan *et al.* [6], who demonstrated that a short-term recovery period or rest interval introduced between load cycles significantly enhances bone formation. Recently, Matsumoto *et al.* developed a noninvasive 3-point bending loading method with programmed wave forms for use on rat tibiae, and observed a relationship between bone formation and mechanical loading [7].

These methods mainly caused woven bone formation on bone surface as a primary response to the applied loads. The mechanical strain must be signaled to the bone surface, i.e., periosteum which is assumed to be responsible for sensing the mechanical strain. The periosteum is a membrane that lines the outer surface of all bones, except at the joints of long bones. This membrane, which consists of dense irregular connective tissue, is divided into an outer fibrous layer and an inner osteogenic layer. The fibrous layer contains fibroblasts, whereas the osteogenic layer contains the progenitor cells that develop into osteoblasts. In the observation of molecular and cellular phenomena acted by mechanical stress *in vitro*, the mechanical stress causes remodeling of cell-matrix adhesions, in which the cytoskeleton rapidly responds to external force by actin assembly [8]. However *in vivo*, although woven bone and lamellar bone formation is enhanced by mechanical loading on bone surface [9], the histological change and action of periosteal cells during bone formation are not fully understood.

In the present study, in order to investigate the cellular and molecular mechanism of mechanotransduction in the mechanically loaded periosteum, developed noninvasive 3-point bending loads with programmed waveforms were applied to mouse tibiae. Since programmed waveforms consist of 2 types, i.e., short-pulse and long-pulse, we selected the latter as giving the less inflammatory signals. After producing a load on the mouse tibial periosteum, we observed hypertrophy of the periosteum. In the hypertrophic periosteum, cell proliferation and osteoblast activation were detected. To investigate the relationship between programmed loading and periosteal hypertrophy, we further devised a new observation method named *in-situ* fluorescence imaging. This imaging method enabled us to observe the quick changes in periosteal cell shapes by mechanical loading. Upon detailed analysis, we observed that the mechanical loading rapidly decreased the quantity of stress fibers of the actin cytoskeleton and changed the nuclear shapes in the periosteal cells, and then disorganized actin cytoskeleton was remodeled in a time-dependent manner. Moreover, by treatment with cytochalasin-D, which disrupts the actin cytoskeleton, periosteal hypertrophy was induced. Furthermore, by intravenously injecting with a ROCK inhibitor, which inhibits polymerization of the actin cytoskeleton, the mechanical loading-mediated periosteal hypertrophy was reduced. Taken together, we demonstrated that actin disorganization and subsequent remodeling were linked to the mechanical stress-mediated hypertrophy of the periosteum, which promoted bone formation.

## Results

### Induction of bone formation in mouse tibia by mechanical loading

A 3-point bending bone formation experimental system was established earlier for the rat tibia [7], providing important knowledge about the new bone formation induced by mechanical loading. In this method, interestingly, we found the new bone formation area at the side opposite to the loading point as well as around the loading point. This previous report suggested that the new bone formation was promoted on the periosteal surface subjected to the stress change toward compression. However, the mechanism of the response of the osseous tissues against the mechanically loaded stress change toward compression has not been well investigated.

**Figure 1:**
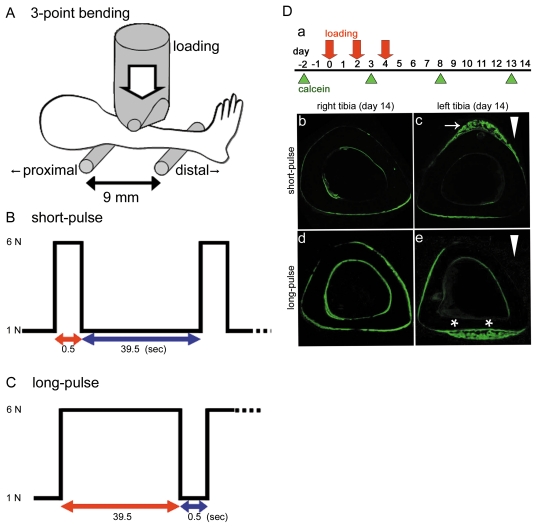
Experimental condition and fluorescent image of tibial sections. (A) Schematic view of 3-point-bending method. The lateral surface of tibia is supported by 2 rubber-padded points set 9 mm apart. The load cell applies the medial surface of tibia in the point set 3.6 mm distal from the proximal supporting point. In the experiment, a static pre-load is applied to the tibia. This load was set to be approximately 1 newton (N). (B, C) Designed wave forms of the loads applied to the mouse left tibia. (B) The short-pulse mode consists of a constant load (0.5 s) and a rest period (39.5 s). (C) The long-pulse mode consists of a much longer period of constant load (39.5 s) and a much shorter rest period (0.5 s). A bout of the mechanical loading comprises 36 cycles lasting a total of 24 minutes. No loads are applied to the right tibia. The magnitude of each load is approximately 6 N. In the experiment, a static pre-load is applied to the tibia. This load is set to be approximately 1 N. (D) Time schedule and fluorescent image of tibial sections (a) The experimental design for the loading and calcein labeling. Loading was performed 3 times (red arrows). Calcein labeling was carried out 4 times (green triangles). (b–e) Calcein fluorescent images of tibial sections (green colored) were examined 14 days after loading. (b, d) In the right tibia, used as a control, new bone formation was not observed in either short-pulse or long-pulse group. (c) In the left tibia in the short-pulse group, bone formation was prominent on the periosteal surface around the loading point (white arrow). (e) In the left tibia in the long-pulse group, bone formation was significant on the periosteal surface at the side opposite to the loading point (white asterisks), but not around the loading point. White arrowheads indicate loading direction.

As shown in Fig. 1A, we adapted this system to 10-week-old male C57BL/6 mice. In the search of the mouse strain adapting the mechanical stress experiment, the next item is important; The mouse attains skeletal maturity at a comparatively early age. Peak long bone strength is attained by 20 weeks in the C57BL/6 strain [10]. Moreover, C57BL/6 is utilized for generation of knockout mice.

The C57BL/6 mice were divided into 2 groups. The first group received a loading, with the waveform designed as illustrated in Fig. 1B, to the left tibia. The waveform of this loading is composed of a constant load (0.5 s) and a rest period (39.5 s), termed the short pulse, which was previously termed as the pulse-load [7]. The second group received a loading with the waveform shown in Fig. 1C, also to the left tibia. This wave form is composed of the constant load (39.5 s) and the rest period (0.5 s), which termed the pulse-unload [7], but we called here “the long-pulse”. A bout of the mechanical loading comprised 36 cycles lasting 24 minutes. The right tibia did not receive a load. The magnitude of each load was 6 newtons (N). The load-magnitude dependent bone formation in the mouse model was previously reported [10]. According this literature, the magnitude of strain is in the range of strain between the first locomotion (approx. 1600 µε) and the landing following a fall from a height of 20 cm (approx. 2600 µε). Hence, we applied the load of 6N to induce this level of strains in the bone. We monitored the actual load cell output in *in-vivo* experiments for the short-pulse and long-pulse mode. The strain induced in the mouse tibia by the dynamic loading was measured with strain gauges. The peak strain on the surface around the loading point was approximately 2100 µε. This strain was equivalent to that observed in the previous investigations on mechanically induced bone formation using mouse model [11].

We show the time schedule for loading (red arrow) and calcein labeling (green triangle) in Fig. 1Da. To identify the new bone formation, we injected calcein 4 times into the abdominal cavity of mice at day −2, 3, 8, and 13 before and after the loading performed 3 times, i.e., at day 0, 2, and 4, by the short-pulse or long-pulse mode. These mice were dissected, and calcein fluorescent images of tibial sections were examined at day 14. In the short-pulse or long-pulse treated left tibia, woven bone formation was prominent around the loading point or at the side opposite to the loading point on the periosteal surface (Fig. 1Dc,e); whereas no significant bone formation was observed in the right tibia (Fig. 1Db,d). In the long-pulse treated group, the bone surface at the side opposite to the loading point was extended during loading (Fig. 1De). Afterwards, during the period of unloading, this bone surface was subjected to change of the stress toward compression.

We also assessed the level of inflammation caused by the loading, by examining the expression of Mac-3, which is a marker of the macrophages that penetrate into inflamed tissue (Fig. S1). In the short-pulse treated group at day 3 around the loading point, strong Mac-3 signals were observed, indicating that a high number of macrophages had penetrated the area, compared with the number on the opposite side in the long-pulse treated group. We then examined the difference in inflammation level between the area around the loading point and the side opposite to it in the long-pulse treated group, and observed a higher number of Mac-3-positive macrophages around the loading point but not at the side opposite to the loading point. Although the inflammation response was thought to affect the bone formation, these results show that the inflammation is not a major factor of this method as to the mechanical loading mediated bone formation, because they show that the side opposite to the loading point was much less inflamed, so-called “noninvasive,” compared with the area around the loading point. Therefore, to investigate the molecular system of mechanotransduction excluding the effects of inflammation, we focused further examination on the opposite side in the long-pulse treated group.

### The stress change toward compression on periosteum induces its hypertrophy

**Figure 2:**
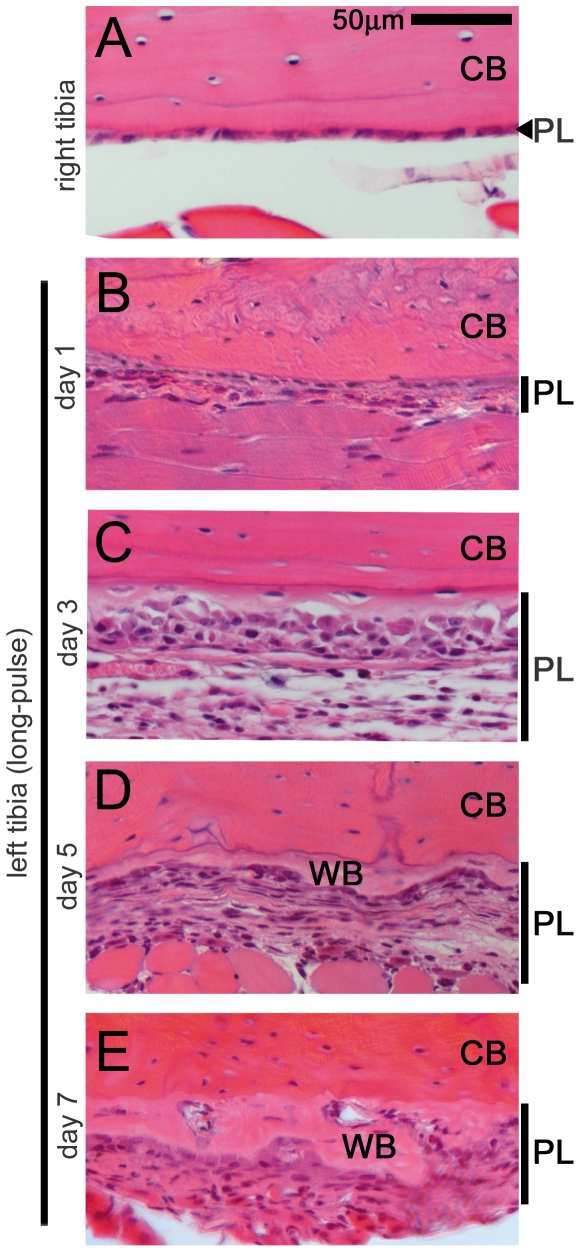
Stress induced in the long-pulse mode induced cell proliferation in the periosteum. Hematoxylin and eosin (H&E)-stained histological sections of the side opposite to the loading point in the long-pulse group at days 1, 3, 5, and 7 after loading to the left tibia. In the right tibia, used as the control, the periosteum consisted of a thin layer (A). In the left tibia treated in the long-pulse mode, periosteal hypertrophy (purple colored nuclei) first occurred at day 1 (B), and became prominent at day 3 (C). The woven bone (pale pink color) first appeared at day 5 (D) and became prominent at day 7 (E). CB, cortical bone; PL, periosteal cell layer; WB, woven bone; Scale bar, 50 µm. A representative result from 4 individual experiments is shown.

To investigate the effects of the stress change toward compression on the periosteum, we histologically analyzed this outer layer of bone. After loading had been applied to the left tibia in the long-pulse condition, we performed hematoxylin-eosin (H&E) staining of the left tibia at days 1,3,5, and 7 (Fig. 2B–E), and of a control unloaded right tibia (Fig. 2A), and found that hypertrophy was present at day 3 and 5. Moreover, the woven bone formation had started at day 5 and became prominent at day 7. These results suggest that the stress change toward compression in the long-pulse mode triggered cell proliferation at the side opposite to the loading point in the periosteum, subsequently resulting in the woven bone formation.

**Figure 3:**
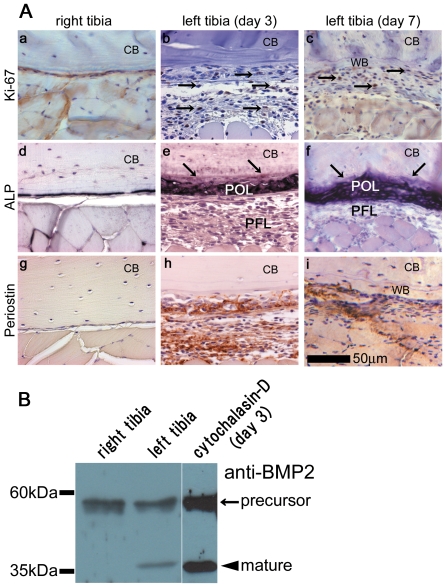
Induction of hypertrophic periosteum by the long-pulse mode. (A) Immunological staining after treatment in the long-pulse mode. Histological sections around the side opposite to the loading point of the long-pulse treated tibiae were prepared at days 1, 3, and 7. These sections were stained with anti-Ki-67 antibodies (a–c) and anti-periostin antibodies (g–i), and ALP activity was also assessed (d–f). In the right tibia, taken as the control, Ki-67-positive cells were rarely detected (a), low ALP activity was observed (d), and restricted periostin signals were observed (g). In the left tibia treated in the long-pulse mode, Ki-67-positive cells were detected at days 3 and 7 (b, c, arrows), and high ALP activity was detected in the osteogenic layer, but not in the fibrous layer, at days 3 and 7 (e, f, arrows). Expression of periostin was detected throughout the hypertrophic periosteum at days 3 and 7 (h, i). CB, cortical bone; POL, periosteal osteogenic layer; PFL, periosteal fibrous layer; WB, woven bone. Scale bar, 50 µm. A representative result from 4 independent experiments is shown. (B) Increased BMP2 protein expression in hypertrophic periosteal cells. Lysates of periosteal cells around the side opposite to the loading point from the loaded left tibia in the long-pulse mode and the control right tibia at day 3 were separated by SDS-PAGE and blotted with anti-BMP-2 antibodies. The arrow indicates precursor BMP-2 bands, and the arrowhead indicates the mature BMP-2 band. The active mature BMP-2 was preferentially expressed in the loaded left tibia, whereas equivalent signals of the precursor BMP-2 were detected for both right and left tibiae. Moreover, in the cytochalasin-D-treated left tibia 3 days after the treatment as shown in Fig. 7, active mature BMP-2 was also significantly expressed.

To confirm the cell proliferation in the long-pulse mode, we performed immunostaining using an anti-Ki-67 antibody, since Ki-67 is a marker of proliferating cells. In the loaded left tibia, Ki-67-positive cells were detected at the side opposite to the loading point treated with the long-pulse at day 3 (Fig. 3Ab), and became more obvious at day 7 (Fig. 3Ac). In the control right tibia, Ki-67-positive cells were rarely detected (Fig. 3Aa). These results indicate that the stress change toward compression induced cell proliferation in the periosteum, leading to hypertrophy.

Next, to identify osteoblasts in the hypertrophic periosteum, we performed staining for alkaline phosphatase (ALP), a marker of osteoblasts. Although ALP is a ubiquitous plasma membrane-bound enzyme synthesized at various levels by many cell types, high levels of ALP are found in osteoblasts and preosteoblasts in bone [12]. Strong ALP positive signals were observed inside the hypertrophic periosteum of the left tibia at day 3 at the side opposite to the loading point (Fig. 3Ae), and became more intense at day 7 (Fig. 3Af); whereas in the control right tibia, very low activity of ALP was observed (Fig. 3Ad). These results indicate that the stress change toward compression increased the number of osteoblasts in the hypertrophic periosteum. In addition, to identify the character of the hypertrophic periosteum, we performed anti-periostin antibody staining, since periostin is a typical marker of periosteum [13], though it is also expressed in pathological tissues like epidermis in wound healing [14]. At day 3, we found periostin to be expressed throughout the side opposite to the loading point in hypertrophic periosteum (Fig. 3Ah); and this signal had decreased in intensity at day 7 because of the reduced area of periosteum by woven bone formation (Fig. 3Ai). In the control right tibia, the expression of periostin was restricted to the thin layer of periosteum (Fig. 3Ag). These results confirm that the stress change toward compression induced the hypertrophy of the periosteum and enhanced periosteal cell proliferation.

### Increase of mature BMP2 protein expression in hypertrophic periosteal cells

Since we observed woven bone formation in response to the stress change toward compression, we investigated the induction of osteoblast differentiation in periosteal cells by analyzing the expression of BMP-2, which is a typical differentiation factor involved in the differentiation of mesenchymal cells into osteoblasts. We performed Western blot analysis on lysates of periosteal cells taken from the side opposite to the loading point from the long-pulse treated left tibia and the control right tibia at day 3. Anti-BMP-2 antibodies detected bands of a precursor of BMP-2 (Fig. 3B, arrow) and a mature BMP-2 (Fig. 3B, arrowhead). The active mature BMP-2 was preferentially expressed in the long-pulse treated left tibia, whereas the signals of the precursor BMP-2 were equivalent between right and left tibia. These results indicate that osteoblast differentiation was induced by the stress change toward compression.

### *In situ* immunofluorescence of cytoskeleton in periosteum of mice by laser-scanning confocal microscope

**Figure 4:**
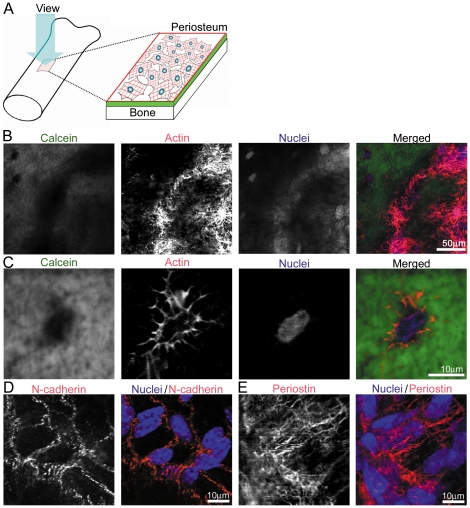
*in-situ* immunofluorescence imaging method. (A) Schematic view of *in-situ* immunofluorescence imaging method for analyzing the periosteum. The calcein-labeled bone surface and immunostained perisoteum were examined by laser-scanning confocal microscope. (B) Immunostained periosteal cells were distinguished from the calcein-labeled bone surface. Periosteal cells were stained with Alexa Fluor 568-labeled phalloidin and TOPRO3 for detecting the actin cytoskeleton and nuclei, respectively; and a merged image is shown in the right column. (C) Osteocytes, embedded in the calcein-labeled bone, were stained with phalloidin for actin and TOPRO3 for nuclei; and a merged image is shown in the right column. (D) Periosteal cells were stained with anti-N-cadherin antibodies, and this photo was merged with that of TOPRO3-labeled nuclei. (E) Periosteal cells were stained with anti-periostin antibodies, and this photo was merged with that of TOPRO3-stained nuclei.

To investigate the molecular and cellular mechanism of mechanotransduction, we developed a new method, *in situ* immunofluorescence imaging, to visualize the rapid appearance of molecules and quick cytoskeletal alterations in the periosteal cells. The schematic of this method is shown in Fig. 4A, which method has the advantage of allowing quick observation *in vivo*. We excised tibiae of mice injected intraperitoneally with calcein 2 days before sacrifice, fixed the whole tibiae, and then immunofluorescently stained them. The calcein labeling on the bone surface enabled us to distinguish the periosteum from the other connective tissues. Firstly, we observed the cytoskeletal actin network in periosteal cells on the bone surface (Fig. 4B), then in the cells embedded just beneath the calcein-labeled bone surface, indicating osteocytes (Fig. 4C). Cytoskeletal actin and nuclei were stained with Alexa Fluor 568-labeled phalloidin and TOPRO3, respectively, showing actin stress fibers on the bone surface. Next, the cell-cell adhesion structure was demonstrated by staining with anti-N-cadherin antibodies, which is a representative mesenchymal cadherin [15], to show adherence junctions in the cell-cell boundary (Fig. 4D). To confirm the presence of periosteum on the bone surface, we stained whole tibia with anti-periostin antibodies [16], resulting in the detection of immunofluorescent signals for periostin in the tissue on the bone surface (Fig. 4E). Thus, we demonstrated that our developed method could visualize possible alteration of cytoskeletal structure and proteins in the periosteum.

### Disorganization and remodeling of actin cytoskeleton triggered by mechanical loading

**Figure 5:**
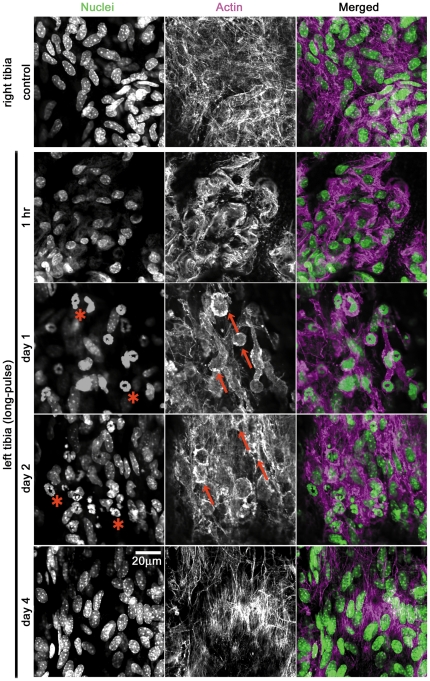
Disorganization and remodeling of actin cytoskeleton in periosteal cells by mechanical loading. Periosteal cells subjected to mechanical loading in the long-pulse mode were stained with Alexa Fluor 568-labeled phalloidin for actin cytoskeleton and TOPRO3 for nuclei one hour after onset of the mechanical loading. The right tibia was used as the unloaded control sample. On the bone surface of the right tibia, the actin cytoskeleton was detected as having the shape of stress fibers (right tibia, control). On the side opposite to loading point of the left tibia treated in the long-pulse mode, the signals of actin stress fibers were decreased in intensity, and these fibers were disorganized (left tibia, 1 hr). Following the first observation after 1 hr in the long-pulse mode, we continuously manipulated this mode to left tibia for 4 days. Periosteal cells in the left tibia subjected to mechanical loading in the long-pulse mode were stained similarly at day 1, day 2 and day 4 with Alexa Fluor 568-labeled phalloidin for actin cytoskeleton and TOPRO3 for nuclei, and both signals were merged. At day 1, actin stress fibers were rarely detected, and round-shaped cells were observed (red arrows). At day 2, actin stress fibers were partially restored (red arrows). Interestingly, nuclei with holes and abnormally shaped nuclei were detected at days 1 and 2 (red asterisks). Disorganized actin stress fibers and abnormally shaped nuclei disappeared at day 4. Scale bar, 20 µm. For all groups, n = 6.

To investigate the immediate effect of periosteal cells right after the mechanical loading, we observed periosteal cells with our developed imaging method. Right after loading to the left tibia in the long-pulse mode, we fixed the mice immediately by injecting them with paraformaldehyde solution via the left ventricle, and then excised the loaded left and the control right tibiae. These tibiae were immunostained, which was observed with our imaging method. On the bone surface of the control right tibia corresponding to the side opposite to the loading point, the actin cytoskeleton was detected as stress fibers (Actin in Fig. 5, right tibia, control); whereas at the same site in the long-pulse treated bone in a manner of quick observation: one hour after onset of treatment, actin stress fibers were decreased in number, and aggregated fluorescent signals for cytoskeletal actin were detected (Fig. 5, left tibia:1 hr). However, at the same site in the short-pulse treated bone, decrease and aggregation of actin stress fibers were rarely detected (data not shown). These results indicate that the stress change toward compression induced quick actin cytoskeletal disorganization at the side opposite to the loading point in the long-pulse mode. Following disorganization, we observed remodeling of the actin cytoskeleton and a change in the nuclear shape of the periosteal cells after the mechanical loading. To investigate actin, after loading to the left tibia in the long-pulse mode, we observed the periosteal cells in the time-dependent manner at day 1, 2 and 4. One day after the long-pulse mode treatment, actin stress fibers were rarely detected in the periosteal cells, which appeared to be round-shaped (Fig. 5, day 1, arrows), and nuclei with a hole in their middle were observed (Fig. 5, day 1, asterisks). Two days after loading, intact actin stress fibers were partially restored in the periosteal cells spreading throughout the periosteum (Fig. 5, day 2, arrows); and abnormally shaped nuclei were observed (Fig. 5, day 2, asterisks). Four days after loading, actin stress fibers and nuclear shapes were recovered (Fig. 5, day 4), compared with the bone surface on non-treated control right tibia. Thus, these results show that the non-inflammatory re-modeling of the actin cytoskeleton occurred in periosteal cells. Taken together, actin disorganization and subsequent re-modeling were thought to be involved in periosteal hypertrophy in the mechanotransduction.

### Induction of periosteal hypertrophy by injection of cytochalasin-D

**Figure 6:**
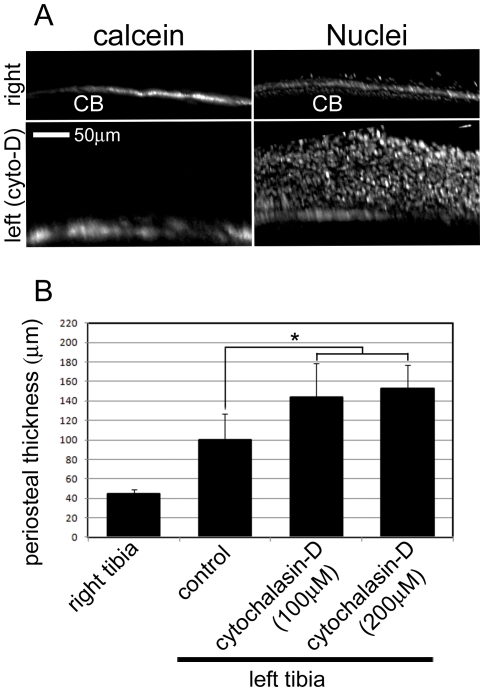
Induction of periosteal hypertrophy by treatment of cytochalasin-D. Periosteal hypertrophy was induced by injection of cytochalasin-D into the periosteum. (A) Periosteal hypertrophy was revealed by observing three-dimensional reconstructed immunofluorescent images. In the control right tibia (right), the calcein-labeled layer (calcein) and a TOPRO3-labeled thin periosteal cell layer (nuclei) were detected. In the 200 µM cytochalasin-D-treated left tibia (left, cyto-D), the calcein-labeled layer (calcein) was again seen, but the hypertrophic periosteal cell layer (nuclei) had become markedly hypertrophic. (B) Thickness of periosteum treated with cytochalasin-D. Thickness was measured from the calcein-labeled bone surface to the superficial aspect of the layer of TOPRO3-labeled nuclei. In a non-treated right tibia, the periosteal thickness was approximately 40 µm (right tibia). In the left tibia treated with DMSO in PBS, the periosteal thickness was about 100 µm (control). In the cytochalasin-D-treated left tibia, the periosteal thickness was increased to about 140 µm (100 µM) and 150 µm (200 µM), respectively. Periosteal thickness was measured by laser scanning confocal microscope (Olympus). CB; cortical bone. For all groups, n = 4. Error bars represent standard deviation. Significant difference (indicated by asterisk, paired t-test) are detected in periosteal thickness between the cytochalasin-D treated groups (100 µM+200 µM, n = 8) and the control (*p*<0.05).

To further investigate whether actin disorganization induced periosteal hypertrophy, we used cytochalasin-D, a fungal metabolite that has the ability to disrupt actin fibers by binding to actin filaments [17]. First, in order to perform local treatment of cytochalasin-D, we dissected the leg skin and fascia for exposure of the tibial periosteum, and treated cytochalasin-D in PBS or DMSO in PBS as a vehicle-treated control into the space between muscles in the periosteum, because cytochalasin-D is toxic, thus it is not injected systemically. The treatment was performed for 5 minutes at room temperature. Soon afterwards, treated periosteum was washed with PBS, and the dissected skin was sutured. 2 days after the treatment, we excised tibia, and immunostainned them with TOPRO3 for nuclei. Thus we observed periosteal hypertrophy with a laser-scanning confocal microscope. To measure the periosteal thickness vertical to the calcein-labeled bone surface, we constructed three-dimensional immunofluorescence images by the superposition of planar immunofluorescence images. We measured the most hypertrophic areas on the treated bone surface. A represent image is shown in Fig. 6A. In the non-treated right tibia, the calcein-labeled layer and the TOPRO3-labeled thin periosteal cell layer were detected. However, in the 200 µM cytochalasin-D-treated left tibia, the calcein-labeled layer and the hypertrophic periosteal cell layer were preferentially detected. The thickness was measured from the calcein-labeled bone surface to the TOPRO3-labeled nuclei. In the non-treated right tibia of mice, the periosteal thickness was approximately 40 µm. In the vehicle-treated left tibia (control), this thickness became about 100 µm, since during the operation on a vehicle-treated control mice, the surgical stimulus seemed to induce periosteal hypertrophy. In the 100 µM and 200 µM cytochalasin-D-treated left tibia, the periosteal thickness was increased to about 140 µm and 150 µm, respectively (Fig. 6B). These results indicate that actin disorganization apparently induced proliferation of periosteal cells.

Moreover, in the Western blot analysis, anti-BMP-2 antibodies revealed that the active mature BMP-2 was preferentially expressed in the cytochalasin-D-treated left tibia 3 days after the treatment (Fig. 3, cytochalasin-D (day 3)).

### Inhibition of remodeling of the actin cytoskeleton suppressed hypertrophy of the periosteum

**Figure 7:**
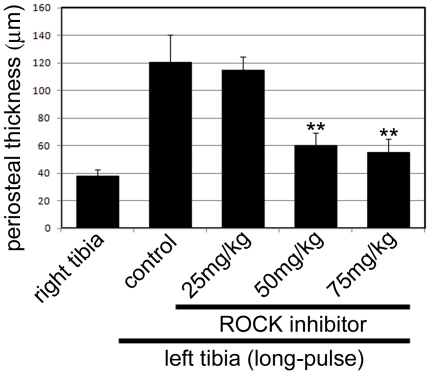
Suppression of periosteal hypertrophy by inhibition of remodeling of actin cytoskeleton by ROCK inhibitor. During the mechanical loading to the left tibia in the long-pulse mode, a ROCK inhibitor, which blocks the actin polymerization in actin remodeling, was injected into mice via a tail vein. The thickness of periosteum was measured at day 3 post loading. In unloaded and non-injected right tibia, the periosteal thickness was about 40 µm. In the loaded and vehicle-injected left tibia, the periosteal thickness was approximately 120 µm (0 mg/kg). In the loaded and ROCK inhibitor-injected left tibia, the periosteal thickness was decreased in a dose-dependent manner (25 mg/kg, 50 mg/kg, 75 mg/kg). Periosteal thickness was measured by observation with a laser scanning confocal microscope (Olympus). For all groups, n = 5. Error bars represent standard deviation. *Significant deference versus non-injected control (0 mg/kg) (*p*<0.05).

Next, to investigate the effect of actin remodeling for periosteal hypertrophy, we injected a ROCK inhibitor that inhibits the Rho-ROCK signal indispensable for the polymerization of actin fibers. Importantly, this inhibitor does not destroy intact actin fibers, but inhibits actin polymerization for remodeling. In the long-pulse mode, mice were loaded twice (day 1 and day 3); and the ROCK inhibitor or the isotonic sodium chloride solution as a vehicle-injected control was injected 4 times (at days 1–4) via a tail vein. The mice were then sacrificed at day 5, and observed for periosteal thickness in the same manner described above. In the vehicle-injected mice, the periosteal thickness was about 120 µm. The results showed that the thickness of the periosteum was slightly decreased in mice injected with 25 mg/kg of the ROCK inhibitor (Fig. 7). However, in 50 mg/kg and 75 mg/kg ROCK inhibitor-injected mice, the periosteum thickness was only one-half of the vehicle-injected control. These results suggest that actin remodeling was important for hypertrophy of the periosteum.

## Discussion

In development of researches for mechanical stress, there was a great need for noninvasive *in vivo* models of mechanotransduction. In this regard, the non-invasive loading on bones of experimental animals, axial compression [3], or bending [2] has been developed and utilized. In the present study, we adopted the bending method considering the effect, which enabled us to apply a large load to bone. In the bending method, the loading could be applied with either 4-point, 3-point or cantilever bending. In the 4-point bending model [2], however, sham loading that did not generate bending stress induced significant bone formation on the periosteal surface [3], [9], because the excessive magnitude of direct pressure in this model is suspected to be responsible for this bone formation. On the other hand, the 3-point bending model [18] or cantilever model [19] requires smaller loading magnitudes, and thus might be useful for the analysis of periosteal bone formation. Hence in this study, 3-point bending was employed. Actually, the loading applied in a 3-point bending was lower than that in a 4-point bending, and the static loading control did not demonstrate the enhanced bone formation [7]. To investigate the mechanism of mechanotransduction, we established 2 systems, the short-pulse and the long-pulse mode, of mechanical loading for rat, which was then applied to mouse tibia. Specifically, in the long-pulse mode, the bone surface at the side opposite to the loading was extended, and during the period of unloading, this bone surface is subjected to change of the stress toward compression, indicating that the long-pulse is much less inflamed or noninvasive. To further demonstrate the signals of mechanotransduction, a new method is required to observe cell morphology in the initial stage of burden of mechanical stress. In this requirement, we developed *in-situ* immunofluorescence imaging.

With coupling the new bending method, the long-pulse mode, with *in-situ* immunofluorescence imaging, we have succeeded in noninvasive loading and quick observation of the periosteum. The results showed that the actin fibers were disorganized soon after loading, and the disorganized actin fibers were remodeled 2 days after loading to induce periosteal cell proliferation. This periosteal hypertrophy induced by cell proliferation led to the formation of woven bone. Since the tissue change in the periosteum after loading was very quick, our 2 newly developed methods were very useful to detect molecular changes without the interference of inflammation. Nevertheless, it was very difficult to catch the first actin disorganization inducing event triggered by the loading. The organization of the actin cytoskeleton may be part of the sensing apparatus used by cells to detect and respond to mechanical signals. *In vitro*, reorganization of the actin cytoskeleton in osteoblasts into stress fiber bundles is required for fluid shear-induced expression of genes, cyclooxygenase-2 and c-fos, linked to mechanotransduction in bone formation [20], [21]. Moreover, during actin disorganization, cofilin can recognize tilt and twist actin to bind it, suggesting that certain actin-binding proteins have evolved to regulate the internal dynamics as part of cellular control of the cytoskeleton [22].

A specific inhibitor of Rho-dependent kinase (ROCK) suppressed periosteal hypertrophy by inhibiting action reorganization for remodeling. On the other hand, while active RhoA is required for stress fiber formation, its principal downstream effectors are the diaphanous-related formin protein mDia 1 that promotes assembly of actin stress fibers, and ROCK that induces non-muscle myosin II-driven actin contraction [8], [23]. Thus, it is interesting to find the link between actin reorganization and actin contraction. It is reported that mechanical stress acting on cell-matrix adhesions causes rapid or sustained changes in RhoA activity [24]. These changes in RhoA or probably ROCK in response to external mechanical stimulation will lead to a complex remodeling of the actin cytoskeleton as well as of cell-matrix adhesions like tenascin-C [25] that is also expressed in periosteal cells [13]. Our in-vivo model of mechanical stress coupled with a rapid analyzing system gives the opportunity to investigate signals in the upstream and downstream of ROCK by manipulating inhibitors of signal transduction.

Interestingly, we observed alteration of the nuclear shape in the early time of loading (from day 0 to 2). Such abnormal shapes of nuclei were reported to be seen in the Hutchinson-Gilford progeria syndrome (HGPS), which is caused by mutations of the lamin A gene resulting in nuclear stiffness [26]. The nucleus itself has been proposed to act a cellular mechanosensor, with alterations in nuclear shape causing conformational changes in chromatin structure and organization and directly affecting transcriptional regulation. By this machinery, extracellular forces can be transmitted across the cytoskeleton to the nucleus, resulting in intranuclear deformations; and the actin cytoskeleton is thought to provide protrusive and contractile forces and compressive bearing microtubules to from a polarized network allowing organelle and protein movement throughout the cell [17]. In fact, compressive stress induces shape changes in chondrocyte nuclei [27]; and collagen synthesis is strongly correlated with nuclear shape [28]. In view of these reports and the discussions therein, we can propose that the compressive stress rapidly induces disorganization of the actin cytoskeleton and thereby causes alteration of the nuclear shape to induce the cell proliferation.

Actin reorganization into stress fibers is required for the induction of osteogenic cells in *in-vitro* experiments. In our stress change toward compression model, we observed mature BMP-2 expression at day 3 after loading. BMP-2 expression is known to be involved in cortical bone healing [29]. For the maturation of BMP-2, the appearance of proteolytic activity in the ECM is important. Therefore, we plan further investigations to determine how disorganization of actin fibers and nuclear alteration are linked to the appearance of proteinases including matrix metalloproteinases [30]. The use of our established systems combined with gene knock-out mice should prove helpful for such investigations.

Finally, in the aspect of clinical application, decreased fracture toughness in bones from older people is indicative of a change in the quality of bone tissue with age. This reduced bone toughness may partly explain the observation that fracture risk in older people is greater than predicted by the loss of bone mass alone [31]. Periosteal apposition of bone with age serves a biomechanically important function by compensating for reduced tissue properties. Our study may provide the opportunities to restore the reduced bone toughness.

## Materials and Methods

### Mice

In this study, 10-week-old male C57BL/6 mice were used. All animal experiments were performed following The Regulation on animal experimentation of Tokyo Institute of Technology and Tokyo Medical and Dental University. This study was approved by the Animal Care and Use Committee of Tokyo Institute of Technology (permit number: 2009047) and by the Institutional Animal Care and Use Committee of Tokyo Medical and Dental University (permit number: 0070175).

### Antibodies and reagents

Rabbit anti-mouse periostin antibodies were described previously [11], [32]. Rabbit polyclonal anti-Ki-67 antibodies (PRO229, YLEM) and rabbit anti-mouse BMP2 antibodies (polyclonal antibodies H-51, Santa Cruz Biotechnology, [33]), were purchased from the sources indicated. Rabbit anti-mouse N-cadherin antibodies and rat anti-mouse Mac-3 antibodies were obtained from BD Biosciences (Pharmingen). Alexa Fluor 568-labeled goat anti-mouse and anti-rabbit antibodies, as well as Alexa Fluor 568-labeled phalloidin, were procured from Molecular Probes (Invitrogen).

Inhibition of actin remodeling was performed by tail vein injection of mice with (*S*)-4-bromo-5-(2-methylhomopiperazin-1-ylsulfonyl) isoquinoline dihydrochloride, a ROCK inhibitor [34]. This molecule is a specific and efficacious ROCK inhibitor comparable to H1152P [35], [36], [37].

### Loading apparatus

An apparatus for the application of noninvasive 3-point bending load to the rat tibia was developed previously [7]. We newly improved this device for use on mice as shown in Fig. 1A. For application, the lateral surface of the tibia is supported by 2 rubber-padded points set 9 mm apart. The mechanical loading point, which applied medial surface of the bone with a similar point set 3.6 mm distal from the proximal supporting points, thus the span ratio of bending was 2∶3. The loading point consists of a steel cylinder padded with a rubber sheet of approximately 2-mm thickness. Electronic signals for loading are generated by a personal computer equipped with a Digital/Analog converter and fed to an electromagnetic material tester (MMT-250MB-10, Shimadzu Co., Kyoto, Japan). In the animal experiments, the static pre-load of 1 newton (N) was applied to the tibia.

### Evaluation of bone formation

After sacrificing mice that had received mechanical loading and had been injected with calcein (2 mg/ml), their tibiae were dissected, fixed in 70% ethanol for 48 hr, and embedded in resin. Serial non-decalcified transverse sections were prepared with a diamond disc saw (Sege Mikrotome; Leica Microsystems, Wetzlar, Germany), and digital fluorescent images of calcein, 1 mm apart, were obtained by using a confocal laser scanning microscope (LSM5 PASCAL, ZEISS). The images were exported into Image J (NIH) for cropping and linear contrast adjustment.

### Histology and immunohistochemistry

For tissue preparation, mice were sacrificed under anesthesia, fixed with 4% paraformaldehyde (PFA) in PBS by transcardial perfusion. And immediately, the excised their tibiae were immersed in 4% PFA in PBS at 4°C for 20 hr. After a wash with PBS, the samples were decalcified in 500 mM EDTA solution at 4°C for 2 weeks, and then were dehydrated through a graded series of ethanol prior to being embedded in paraffin. Series of 5-µm-thick paraffin sections were prepared for histological analysis or for immunostaining. For immunostaining of Ki-67, the antigen unmasking technique with citrate buffer was performed. For demonstrating the alkaline phosphatase activity, 5-bromo-4-chloro-3-indolylphosphate/nitroblue tetrazoliumchloride (BCIP/NBT) was used as the chromogen.

### *In-situ* immunofluorescence imaging method

Mice were fixed with fresh 4% PFA in PBS as described above. After a wash with PBS, soft tissues were dissected, and the excised whole tibia was incubated with 10 µg/ml glycine in PBS to block free aldehyde groups for 10 min, and then with 0.1% Triton X-100 for 5 min at room temperature. After having been blocked with 1% bovine serum albumin (BSA) in TBS (preheated at 56°C for 30 min) for 30 min at room temperature, the whole tibia was incubated with the desired primary antibodies in 1% BSA in TBS for 2 hr at room temperature. Further incubation with an optimal dilution of fluorescence-tagged secondary antibodies was performed for 1 hr, followed by washing and post-fixation with 4% PFA. Cytoskeletal actin was stained with Alexa Fluor 568-labeled phalloidin. Nuclei were stained with TOPRO3 (Molecular Probes, Invitrogen). Immunostained whole tibiae were immersed into dish. Fluorescent images were collected by a laser-scanning confocal microscope equipped with a water-immersion objective lens (FV1000-BX61, Olympus, Japan). The images were imported into Photoshop (version CS2, Adobe) for cropping and linear contrast adjustment.

### Western blotting

For Western blotting of BMP2, periosteal cells were collected from tibia subjected to mechanical loading or drug injection at day 3. These cells were lysed with SDS sample buffer containing 50 mM dithiothreitol, and the lysates were then boiled for 10 min. Equal amounts of samples were separated on 13% SDS-PAGE gels and then transferred to nitrocellulose transfer membranes. Non-specific binding was blocked by immersion of the membranes for 30 min in 5% skim milk in TBS containing 0.1% Tween 20 (TBST) at room temperature. The membranes were washed with TBST, incubated overnight at 4°C with primary antibodies diluted in 1% skim milk in TBST. After having been washed 3 times with TBST, the membranes were incubated with secondary antibodies for 1 hr at room temperature; thereafter, they were washed 3 times with TBST, incubated with Immobilon Western Chemiluminescent horseradish peroxidase substrate (Millipore), and exposed to Fuji medical x-ray film (Fujifilm Corporation, Tokyo, Japan).

### Statistics

Welch's t tests in combination with Bonferroni-Holm correction for multiple comparisons were performed with the R software (http://www.r-project.org/), in which statistical significance was assumed for *p*<0.05.

## Supporting Information

Figure S1 **Mac-3 staining after treatment in mechanical loading.** Tibial histological sections were prepared at day 3. These sections were stained with anti-Mac-3 antibodies. (A, C) In the right tibia, used as a control, Mac-3-positive cells were rarely detected in either short-pulse or long-pulse group. (B) In the left tibia in the short-pulse group, Mac-3-positive cells were detected around the loading point (arrow). (D, E) In the left tibia in the long-pulse group, Mac-3-positive cells were also detected around the loading point (E, arrow), but not at the side opposite to the loading point (D). Non-specific signals were detected in osteogenic layer. Scale bar; 50 µm (A–D), 200 µm (E). Red arrowhead indicates loading direction.
(TIF)
