# Supplementary figures and images for: Remodeling of Actin Cytoskeleton in Mouse Periosteal Cells under Mechanical Loading Induces Periosteal Cell Proliferation during Bone Formation
Source: PLoS One. 2011 Sep 14;6(9):e24847. doi: 10.1371/journal.pone.0024847 (PMC3173483; doi:10.1371/journal.pone.0024847)

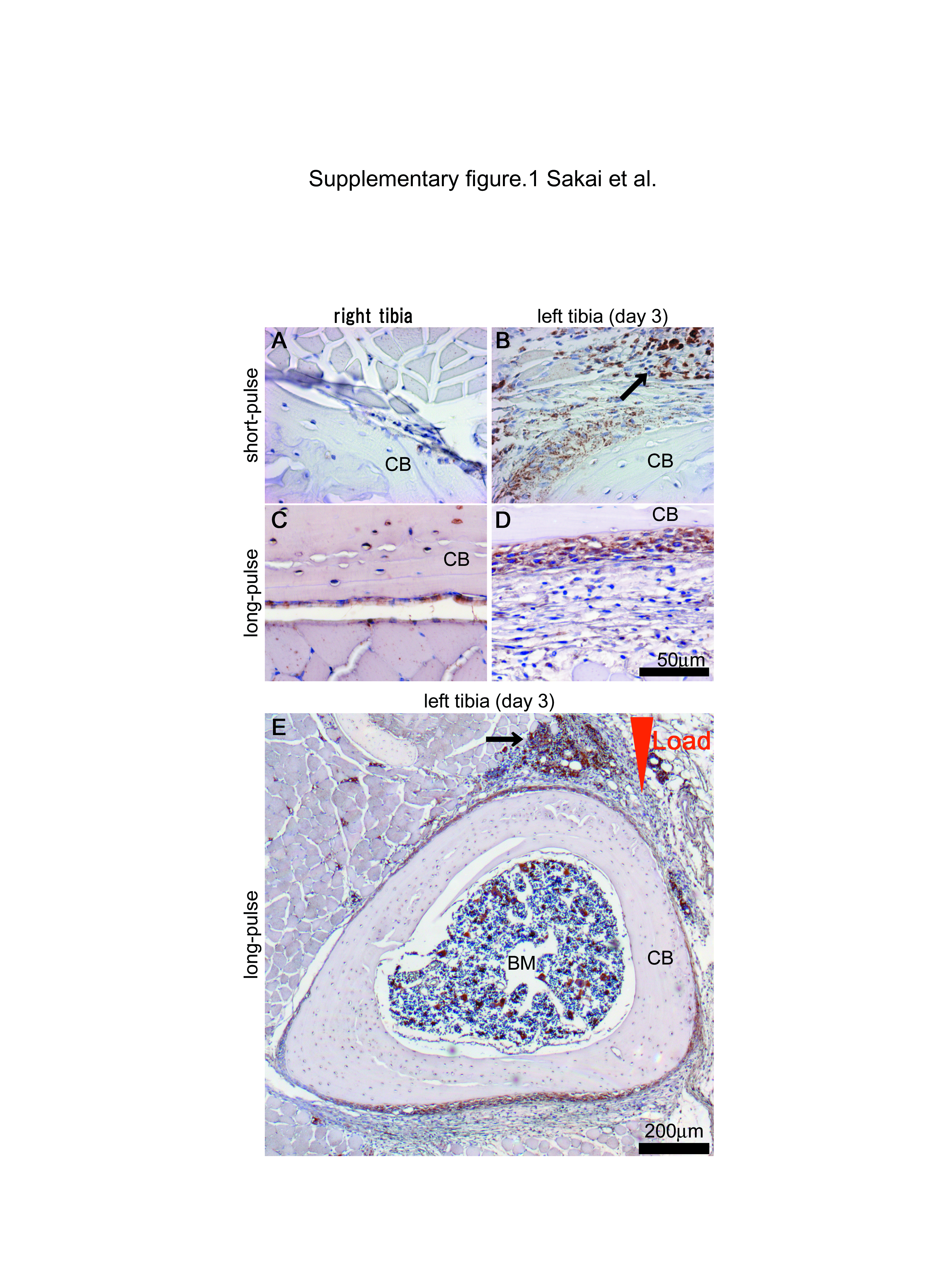

Supplement: Figure S1 — Mac-3 staining after treatment in mechanical loading. Tibial histological sections were prepared at day 3. These sections were stained with anti-Mac-3 antibodies. (A, C) In the right tibia, used as a control, Mac-3-positive cells were rarely detected in either short-pulse or long-pulse group. (B) In the left tibia in the short-pulse group, Mac-3-positive cells were detected around the loading point (arrow). (D, E) In the left tibia in the long-pulse group, Mac-3-positive cells were also detected around the loading point (E, arrow), but not at the side opposite to the loading point (D). Non-specific signals were detected in osteogenic layer. Scale bar; 50 µm (A–D), 200 µm (E). Red arrowhead indicates loading direction. (TIF) [file pone.0024847.s001.tif]
